# Effect of physiological characteristics, smoking, and alcohol use on isoniazid hair drug levels among pulmonary TB patients: a cross-sectional study at one month of intensive TB treatment

**DOI:** 10.4314/ahs.v25i2.2

**Published:** 2025-06

**Authors:** Grace Muzanyi, Muhammad Ntale, Robert Salata, Jackson Mukonzo, David K Mafigiri, Paul Mubiri, Moses Joloba, Godfrey S Bbosa

**Affiliations:** 1 Department of Pharmacology and Therapeutics, Makerere University College of Health Sciences; 2 Uganda Case Western Reserve University Research Collaboration, Uganda; 3 Department of Chemistry, College of Natural Sciences, Makerere University Uganda; 4 Case Western Reserve University Cleveland Ohio, USA; 5 School of Social Sciences, Makerere University College of Humanities and Social Sciences, Uganda; 6 Institute of Public Health Makerere University, College of Health Sciences, Makerere University, Uganda; 7 Department of Medical Microbiology, Makerere University College of Health Sciences, Uganda

**Keywords:** Physiological characteristics, smoking, TB patients, intensive TB treatment

## Abstract

**Background:**

Tuberculosis(TB) is still among the leading causes of death from a single infectious pathogen worldwide. TB treatment is long, requires multiple drug combinations, and therefore adherence monitoring. TB hair drug levels have been suggested as a technique of treatment adherence monitoring; however, the drug levels might be affected by physiological factors, alcohol use, and cigarette smoking. This can affect the pharmacokinetics and pharmacodynamics of TB drugs.

**Objective:**

To assess the effect of physiological factors, smoking, and alcohol on isoniazid hair drug levels during TB treatment.

**Methods:**

Patients were assessed for diabetes Mellitus, smoking, alcohol consumption, age, weight, and gender. Hair drug levels were measured by Liquid Chromatography Mass Spectrometry(LC-MS).

**Results:**

We screened a total of 102 TB patients and enrolled 56 participants out of which 50 completed the study. We excluded 15 participants who were not sampled exactly at 1-month appointment dates and of the remaining 35; the mean hair drug level was 0.0706ng/mg, 95% CI: 0.0303-0.1109. Tests of interaction across the different subgroups yielded no statistically significant interaction coefficients(IC) except for age and gender (IC=-0.36.95%, CI: -0.55—0.17, P=0.001).

**Conclusion:**

Physiological factors, alcohol, and smoking do not affect isoniazid hair drug levels.

## Introduction

TB still ranks among the leading causes of death from a single infectious pathogen worldwide notably in developing countries^1^. The current treatment for TB lasts for at least 6 months and requires adherence monitoring^2^. The World Health Organization (WHO)-recommended directly observed therapy (DOT) for adherence monitoring is logistically challenging in resource-constrained settings like Uganda^3^. Alternative methods of assessing adherence to TB treatment include assessing drug levels in plasma, urine, or saliva but these assess short-term exposure and also pose logistical challenges^4^. Drug concentrations in hair assess long-term exposure and have demonstrated pharmacodynamic relevance in HIV treatment^5^. Currently, hair drug levels are being used to monitor adherence to HIV treatment and there are attempts to extrapolate this to TB treatment^6^. Studies have shown that age, weight, gender, smoking, and alcohol can impact the absorption, body distribution, elimination, and exposure to drugs^7^-^9^. In addition, studies have shown that age is associated with a progressive decline in the functional reserve of body organs and systems, which impacts drug disposition^10^-^11^. Aging is associated with a decline in first-pass metabolism, and therefore bioavailability of certain drugs can be increased^12^. Additionally, increasing age leads to increased body fat and body water and a decreased lean body mass^13^. As a result, hydrophilic drugs have a smaller apparent volume of distribution (V) and lipophilic drugs have an increased V with a prolonged half-life. Drugs with a big hepatic extraction ratio show a degree of age-related decrease in systemic clearance (CL), but for the majority of drugs with a low hepatic extraction ratio, CL is not reduced with advancing age. Obesity is a global problem, with disastrous health, social and economic implications^14^. The computation of drug dosages for obese patients is a challenge, notably for drugs with a narrow therapeutic index. The key factors that affect the tissue distribution of drugs include body composition, regional blood flow, and the affinity of the drug for plasma proteins and/or tissue components. Being obese leads to larger absolute lean body masses as well as fat masses for many individuals. In obese people, the percentage of fat per kg of total body weight (TBW) is markedly increased, whereas that of lean tissue is reduced^15^. Cardiac performance and adipose tissue blood flow are usually altered in obesity^16^.

Gender-associated variations in pharmacokinetics have often been considered important determinants of the clinical effectiveness of drugs^17^. The variations underlying gender-specific pharmacokinetics can be molecular and physiological^8^. Studies show that men might have higher activity compared to women with regard to cytochrome P450 (CYP) isoenzymes CYP1A2 and CYP2E1, for the drug efflux transporter P-glycoprotein, and for isoforms of glucuronosyltransferases and sulfotransferases^18^. Women might have a higher CYP2D6 activity^19^. No gender differences exist for CYP2C^19^ and CYP3A^20^. The often-referenced higher hepatic clearance in women for substrates of CYP3A and P-glycoprotein, e.g. erythromycin may be due to increased intrahepatocellular substrate availability due to lower hepatic P-glycoprotein activity in women relative to men. Physiological factors leading to gender-related pharmacokinetic differences include the perceived lower body weight and organ size, higher amounts of body fat, lower glomerular filtration rate, and different gastric motility in women compared with men. Acute and chronic alcohol consumption can change both the pharmacodynamics and pharmacokinetics of drugs^21^-^23^. The change and mechanism of alcohol kinetic interactions with other drugs can, in part, be on the basis of the binding of the drug to plasma proteins, the ability of the liver to extract the drug from blood passing through the liver, and the distribution space of the drug. Highly bound drugs with low intrinsic hepatic clearance are among the most commonly reported to have their kinetics altered by ethanol (e.g. benzodiazepines, phenytoin, tolbutamide, and warfarin). Less highly bound drugs are less consistently affected. Giving alcohol to laboratory animals inhibits the mixed-function oxidase activity. In humans, the elimination half-life of meprobamate and tolbutamide is increased by acute ethanol administration. Studies have shown that cigarette smoking induces total body drug metabolism, as indicated by decreased antipyrine t½ and increased antipyrine clearance^24^

In a nutshell, studies have shown that factors like gender, age, obesity, smoking, and alcohol consumption do impact drug pharmacokinetics and pharmacodynamics but data on the impact of these factors on hair drug levels of isoniazid is lacking. With the current aim of taking on isoniazid hair drug levels as a tool to monitor adherence to TB treatment, it is prudent to fill this literature gap by generating data to answer this question. It is against this background that a study was conducted to assess the impact of age, gender, smoking, and alcohol consumption on isoniazid hair drug levels.

## Materials and Methods

### Study design

We conducted a cross-sectional study at the end of 1 month of the intensive phase of TB treatment. Patients were screened for diabetes Mellitus, smoking, and alcohol consumption and had their weight measured. Age and gender were compiled through a standard questionnaire. Hair drug levels were measured by LC-MS.

### Ethical review and clearance

This study was approved by the ethics committee of the Makerere University School of Biomedical Sciences (SBS2021-18) and the Uganda National Council for Science and Technology (HS2231ES).

### Study procedures

#### Hair collection procedure

Hair was collected from the occipital region of the scalp using a pair of disposable scissors for each participant. The cut hair was placed on a thin aluminum foil with a thin label taping the distal end of the hair thatch to the foil to mark the directionality of proximal to distal. The specimen was then sealed inside a plastic bag containing a desiccant pellet and stored at room temperature. Hair samples were kept in a dark room prior to transfer to the government analytical laboratory in Wandegeya. Forty (40) strands of hair were collected from each participant at each of the visits: weeks 4, 8, and 26.

#### Hair drug level assay procedure

We developed, calibrated, and standardized the hair drug assay technique using commercially available samples of GMP isoniazid. Human hair samples (3 mg, about 30-40 strands cut to 1cm segments) were pulverized using an Omni bead raptor homogenizer and were extracted in methanol. The extracted samples were then separated on a Synergi Polar-RP (2.1 × 100 mm, 2.5 µm particle size column using water with 0.2% (v/v) formic acid as mobile phase at a flow rate of 0.4 mL/min. The isoniazid levels were monitored at multiple reaction modes using an electrospray ion source operated in positive polarity in an AB Sciex API 5500. Two transitions were used to monitor the INH analyte: 138.1 > 79.0 m/z and 138.1 >120.9 m/z. The analyte was quantified by isotope dilution method using Isoniazid-d4. It had a linear dynamic range of 0.0-0.5 nanograms (ng) INH/milligrams (mg) hair with 2.4-10.8% relative error and precision of 7.5-14.8% coefficient of variation (CV) assessed using three levels of quality control (QC) samples (0.2, 10, 20 ng/mg). An overall recovery rate of 79.5% (with 4.6-7.0% CV) was achieved for all three QC samples.

#### Treatment adherence monitoring procedure

We conducted face-to-face adherence sessions at the initial pretreatment visit and after that at every visit. Treatment was administered 7/7 a week as DOT/SAT. About 27 patients were treated by DOT and 28 were treated by SAT. For those on DOT, drugs were stored at a nearby community drug clinic near the patient's residence and a health worker in this unit was the DOT supervisor. For those on SAT, to enhance the validity of data, we did multiple assessments including pill counts and counting checked doses on the treatment cards.

### The data collection tool

We used a standard history and physical examination form that had been tested, validated, and used in past clinical research studies at the Uganda-Case Research collaboration. It has been used as a standard document for data collection during the past 5 studies at the Uganda-CWRU project clinic. It objectively dug for the patient's history and collected thorough data from physical examination findings by the doctor.

### Data quality control

Data collection, cleaning, and analysis were done stringently in line with the research protocol, SOPS, and applicable regulatory requirements in a clear, justified, timely, original, rigorous, and relevant approach. The data was appraised by assessing its credibility, dependability, conformability, and transferability.

### Data analysis

We cross-tabulated the categorized variables, at the 1-month visit. We computed the mean hair drug levels both overall and per category with the corresponding 95% confidence intervals and p-values. We conducted both univariate and multivariate analysis by using the multivariate linear regression model. We conducted interaction testing using the chi-square test. The multivariate linear regression model was used to estimate the association of the relevant INH levels in hair with each or a combination of the factors; weight, age, gender, alcohol consumption, and smoking.

## Results

**Figure 1:**
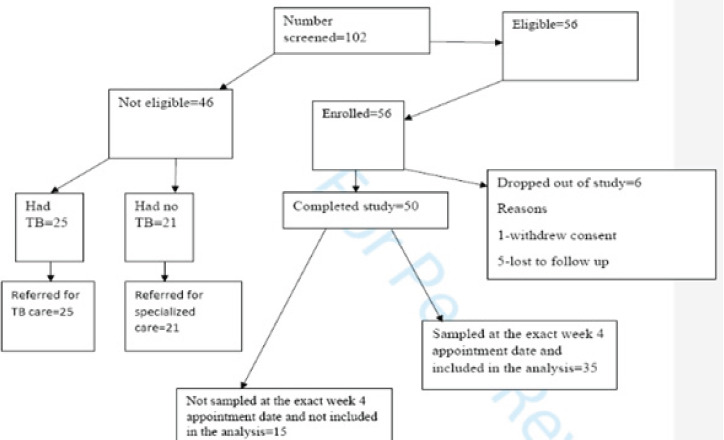
Study profile

From May 2022 through May 2023, we screened 102 TB patients (figure 1) and enrolled 56 participants. There were more males than females and majority of the participants were below age 30 years (Table 1). The results illustrate a mixed group of confirmed TB patients that include smokers, alcohol consumers, and different age categories. The analysis excluded 15 participants who were not sampled exactly at the week 4 appointment dates and of the remaining 35 that were sampled on time; the mean hair drug level was 0.0706ng/mg, 95% CI: 0.0303-0.1109. Tests of interaction between most factors (table 3) yielded statistically insignificant interaction coefficients(IC) except for age and gender which yielded a negative statistically significant correlation coefficient of interaction but this was a very weak interaction (IC=-0.36.95%CI: -0.55—0.17, P=0.001)

**Table 1:** Baseline characteristics of study participants

Variables	Frequency	Percentages(%)
**Gender**		
Male	36	64.29
Female	20	35.71

**Age (Years)**		
<20	7	12.50
21-30	27	48.21
>30-40	13	23.21
>40	9	16.07

**Smoking**		
Yes	4	7.14
No	52	92.86

**Alcohol**		
Yes	5	8.93
No	51	91.07

**Weight (Kgs)**		
<40	0	0.00
>40-50	18	32.14
51-60	23	41.07
>61	15	26.79

### Impact of gender on hair drug levels

Male participants had a higher mean hair drug level (table 2) than their female counterparts, but this did not reach statistical significance after univariate and multivariate analysis.

**Table 2:** Univariate analysis for hair drug levels at the week 4 visit

Variables	Freq	Mean (std Dev)	95% Conf. Interval		P-Value
**Gender**					
Male	23	0.0783 (0.1438)	0.0161	0.1405	
Female	12	0.0559 (0.0281)	0.0381	0.0738	0.6001

**Age (Years)**					
<20	6	0.1575 (0.2719)	-0.1279	0.4428	0.1359
21-30	17	0.0560 (0.0428)	0.0340	0.0780	Ref
>30-40	7	0.0392 (0.0269)	0.0143	0.0641	0.3490
>40	5	0.0603 (0.0453)	0.0041	0.1164	0.8471

**Smoking**					
Yes	3	0.0762 (0.0511)	-0.0507	0.2031	0.9328
No	32	0.0701 (0.1221)	0.0261	0.1141	

**Alcohol**					
Yes	5	0.0677 (0.0493)	0.0065	0.1289	0.9529
No	30	0.0711 (0.1257)	0.0242	0.1180	

**Weight (Kgs)**					
>40-50	12	0.0437 (0.0292)	0.0252	0.0623	0.2186
51-60	12	0.0626 (0.0426)	0.0355	0.0897	Ref
>61	11	0.1087 (0.2033)	-0.0278	0.2453	0.4503

**Table 3:** Multivariate analysis for hair drug levels at week 4

Variables	Interaction Coefficient	Interaction 95% Conf Interval		Interaction P-values
Age + Weight	-0.0481	-0.1109	0.0147	0.128
Age + Gender	-0.3582	-0.5474	-0.1690	0.001
Gender + Weight	0.0441	-0.0652	0.1535	0.416
Age + Alcohol	0.0160	-0.1349	0.1669	0.830
Age + Smoking	-0.0220	-0.1858	0.1417	0.785
Weight + Alcohol	0.0738	-0.2250	0.3726	0.618
Weight + Smoking	-0.1131	-0.4337	0.2075	0.477
Gender + Alcohol	-0.0023	-0.2967	0.2922	0.988
Gender + Smoking	0.0344	-0.2847	0.3535	0.827
Age+weight+gender+alcohol+smoking	0.0086	-0.0508	0.0679	0.768

### Effect of age on Isoniazid hair drug levels

We had 4 age categories with the age bracket 21-30 years being dominant. Across all four age categories, there was a demonstrated presence of isoniazid in the hair of each of these groups as illustrated by the mean hair drug levels (table 2) but overall we found no statistically significant difference between these age groups and the reference group (category 21-30) on univariate and multivariate analysis.

### Effect of smoking on isoniazid hair drug levels

There were more non-smokers than smokers in this study (table 2). We detected isoniazid in the hair of both smokers and non-smokers. We found no statistically significant difference between the mean hair drug levels of smokers compared to non-smokers on univariate and multivariate analysis.

### Effect of alcohol on hair drug levels of isoniazid

We had more non-alcohol consuming participants than the alcohol consumers (table 2). We detected isoniazid in the hair of all alcohol and non-alcohol-consuming participants. In comparison of the alcohol consumers and non-consumers, we found no statistically significant difference between the two groups on univariate and multivariate analysis.

### Calibration output

**Figure 2:**
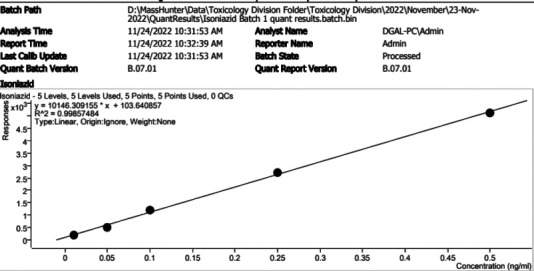
Calibration curve

**Figure 3:**
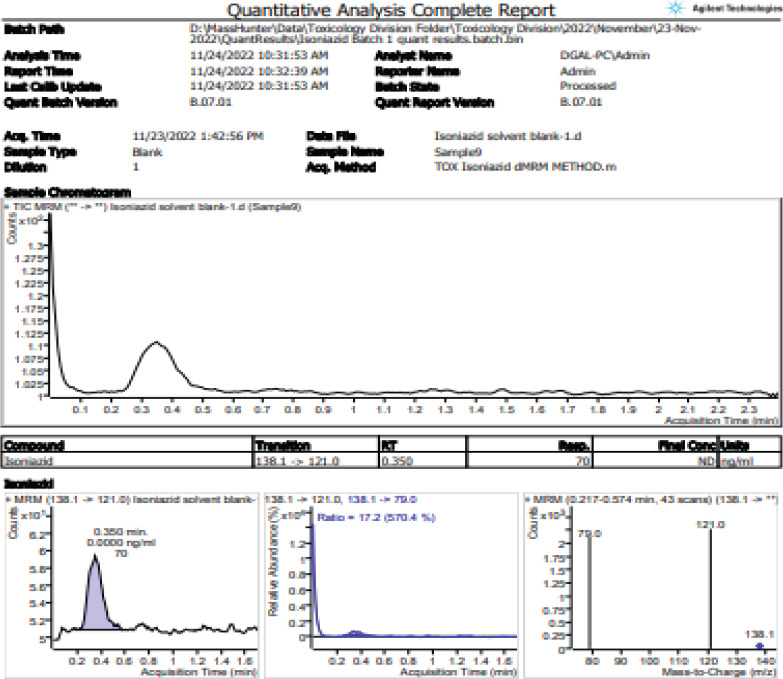
Analysis chromatograms

The study calibration technique yielded a coefficient of determination (R2) of 0.99 with a limit of detection of 0.5ng/ml for five different standardized samples of isoniazid (figure 2). Overall, 90% of the samples run had a clear output of chromatograms (figure 3)

### Effect of weight on isoniazid hair drug levels

We had three weight categories in this analysis. We were able to demonstrate the presence of isoniazid in hair across all three weight categories. There is an increase in the mean isoniazid hair drug level with an increase in weight however; this did not reach statistical significance across all weight categories when compared with univariate and multivariate analysis

## Discussion

We found that age, gender, smoking, alcohol consumption, and weight don't impact isoniazid hair drug levels albeit their impact on plasma levels of different drugs. Our study is the first to assess how these factors impact hair drug levels of anti-TB drugs in an era where hair drug levels are being considered as an alternative to monitor adherence, exposure, and outcomes of TB treatment. Mave et al conducted a study on Measuring TB drug levels in the hair of adults and children to monitor drug exposure and outcomes^25^. He found that hair levels of INH and its metabolite may predict TB treatment outcomes, indicating the potential utility of this measure to assess and optimize TB treatment outcomes however; this study didn't address directly how age can impact isoniazid hair drug levels. Eisenhut et al conducted a study on hair analysis for determination of Isoniazid concentrations and acetylator phenotype during antituberculosis treatment^26^. He found that hair analysis for INH showed lower concentrations in females. Acetyl-INH/INH ratios were significantly lower in patients with slow acetylator versus rapid acetylator genotypes. We found a similar result of lower isoniazid hair drug levels in females compared to males but this was not statistically significant in our study. This might be worthy of further investigation. Weathermon et al's descriptive article assert that several drugs can interact with alcohol, thereby altering the metabolism or effects of alcohol and/or the drugs; however, this article didn't explore how alcohol affects isoniazid hair drug levels as our study did^27^. Margreke J. E. Brill et al descriptive article asserts that the influence of obesity on drug metabolism and elimination greatly differs per specific metabolic or elimination pathway but this article too didn't explore how isoniazid hair drug levels can be affected by body weight^28^. William J. Jusko et al article asserts that cigarette smoking contributes to the considerable variation in inter-individual rates of drug metabolism but this study didn't address our area of interest of how smoking does impact isoniazid hair drug levels^29^.

### The limitations of our study were

1. We didn't test the impact of key factors like diabetes because we enrolled only one participant with diabetes who later withdrew consent. This should be addressed in future studies
2. We didn't test for the impact of the acetylator status on hair drug levels because all the enrolled participants in our study were fast acetylators so we had no comparator group. This should be addressed in future studies

### The strengths of our study are

1. It was conducted in a high TB burden setting; all participants were confirmed TB patients who were on treatment with the first-line TB regimen. Therefore, we were able to collect our data from the right population.

## Conclusion

TB patient factors that include age, gender, weight, smoking, and alcohol consumption do not impact hair drug levels of isoniazid

## Policy recommendation

Tools to use isoniazid TB hair drug levels to monitor adherence, exposure, and TB treatment outcomes should be devised to cut across all age groups, smokers and non-smokers, obese and lean, alcohol-consuming and non-alcohol-consuming TB patients.
